# Incidence, risk factors and clinical outcomes of acute kidney injury after heart transplantation: a retrospective single center study

**DOI:** 10.1186/s13019-020-01351-4

**Published:** 2020-10-07

**Authors:** Yi-Yao Jiang, Xiang-Rong Kong, Fen-Long Xue, Hong-Lei Chen, Wei Zhou, Jun-Wu Chai, Fei Wu, Shan-Shan Jiang, Zhi-Long Li, Kai Wang

**Affiliations:** 1 Department of Cardiovascular Surgery, Tianjin First Center Hospital and NanKai University, Tianjin, China; 2 Department of Cardiovascular Surgery, The First Affiliated Hospital of Bengbu Medical College, Bengbu, Anhui Province China

**Keywords:** Acute kidney injury, Heart transplantation, Mortality, Outcomes

## Abstract

**Objectives:**

This study aimed to identify the incidence rate of Acute kidney injury (AKI) in our center and predict in-hospital mortality and long-term survival after heart transplantation (HTx).

**Methods:**

This single-center, retrospective study from October 2009 and March 2020 analyzed the pre-, intra-, and postoperative characteristics of 95 patients who underwent HTx. AKI was defined according to the Kidney Disease: Improving Global Outcomes (KDIGO) criteria. Risk factors were analyzed by multivariable logistic regression models. The log-rank test was used to compare long-term survival.

**Results:**

Thirty-three (34.7%) patients developed AKI. The mortality in hospital in HTx patients with and without AKI were 21.21 and 6.45%, respectively (*P* < 0.05). Recipients in AKI who required renal replacement therapy (RRT) had a hospital mortality rate of 43.75% compared to 6.45% in those without AKI or RRT (*P <* 0.0001). A long cardiopulmonary bypass (CPB) time (OR:11.393, 95% CI: 2.183 to 59.465, *P* = 0.0039) was positively related to the occurrence of AKI. A high intraoperative urine volume (OR: 0.031, 95% CI: 0.005 to 0.212, *P* = 0.0004) was negatively correlated with AKI. AKI requiring RRT (OR, 11.348; 95% CI, 2.418–53.267, *P* = 0.002) was a risk factor for mortality in hospital. Overall survival in patients without AKI at 1 and 3 years was not different from that in patients with AKI (*P* = 0.096).

**Conclusions:**

AKI is common after HTx. AKI requiring RRT could contribute powerful prognostic information to predict mortality in hospital. A long CPB time and low intraoperative urine volume are associated with the occurrence of AKI.

## Introduction

Heart transplantation (HTx) is a generally successful procedure for patients with end-stage heart failure, improving their survival and quality of life [1]. Acute kidney injury (AKI) is a frequent complication following HTx. With an incidence ranging from 14 to 76%, it is a significant contributor to high morbidity and mortality [2–7]. There are various causes of AKI, such as renal hypoperfusion, prolonged cardiopulmonary bypass (CPB), and nephrotoxicity of immunosuppressive drugs [8]. Therefore, there is a need to identify high-risk factors, reduce the life-threatening outcomes of AKI, and enhance the long-term survival rates in HTx patients.

Scoring systems for the quantification of AKI have been applied in clinical studies. These criteria included the Risk/Injury/Failure/Loss/End-stage (RIFLE) criteria, the Acute Kidney Injury Network (AKIN) criteria, and the Kidney Disease: Improving Global Outcomes (KDIGO) criteria [9–11]. Based on these criteria, AKI can be defined and staged. It is important to note that despite different performances of different scoring systems, a mild to modest form of AKI does not play a significant role in predicting poor long-term outcomes [12]. However, patients who develop severe AKI frequently receive renal replacement therapy (RRT) as a salvage treatment. The need for RRT has been reported to be one of the most important predictors of a poor prognosis after HTx [13]. Although some clinical outcomes associated with AKI requiring RRT have been described, its impact on long-term survival has not been addressed.

The aims of this study were to (1) evaluate the incidence of AKI after HTx in our center by using the KDIGO criteria, (2) identify risk factors for AKI and in-hospital mortality after HTx, and (3) explore long-term survival in AKI patients.

## Methods

### Patient population

Between October 2009 and March 2020, 113 patients were transplanted. According to exclusion criteria, 10 patients were underwent for combined heart-kidney transplant were not included; 8 patients who were lost to follow-up were also excluded. We included 95 patients in the final analysis. All patients were followed in the outpatient department.

### Perioperative management

A biatrial technique was performed in the HTx procedure. All patients received methylprednisolone intraoperatively (500 mg when the aortic cross clamp was released) followed by 120 mg q8h intravenous for the first 24 h, 120 mg q12h for the second 24 h, 120 mg once daily for the third 24 h and basiliximab (20 mg loading dose in the operating room and 20 mg the fourth day). On the fourth day postoperation, tacrolimus and mycophenolate mofetil was started at an oral dose of 1.5 mg and 500 mg twice daily, respectively. Tacrolimus and mycophenolate mofetil were prescribed for the rest of their lives. Further, dosing was based on tacrolimus whole-blood trough concentrations at 6 a.m. (12 h post-dose). A whole-blood tacrolimus trough concentration between 7 and 15 ng/ml was considered in the first 3 months and thereafter tapered towards 5–10 ng/ml. Accompanying immunosuppression comprised corticosteroids, prednisolone was started 28 mg orally on the fourth day postoperation, followed by 24 mg once daily and tapered off to 8 mg once daily orally.

### Outcome measure

AKI was classified according to the KDIGO criteria [11]. The KDIGO criteria recognize 3 stages of AKI severity based on serum creatinine (SCr) levels and urine output. The definition of AKI was based on peak creatinine within 7 days postoperation. The administration of loop diuretics is a commonly used method in the postoperative period and in the early management of AKI, at least in patients with volume overload and/or oliguria. Indications for RRT were stage 3 AKI combined with one of the following: hyperkalemia, severe hypervolemia, uncorrectable metabolic acidosis or serious uremia.

Continuous variables are presented as mean ± standard deviation, while categorical or integer variables are presented as number and percentage. To compare values between two groups, Student’s *t* test was used for normally distributed numerical variables, and Wilcoxon rank test for nonnormally ones. One-way analysis of covariance (ANOVA) or Kruskal-Wallis test was used for comparisons more than two groups. When there was a statistical significance among groups, SNK method was used to perform comparison between groups. Categorical or integer parameters compared by Fisher’s exact test or Chi-square test. Other continuous variables were expressed as median and interquartile (25th to 75th percentile) range and compared by Mann-Whitney U-test or Kruskal-Wallis test. Survival analysis was performed using log-rank test. Cox proportional hazards model were used to identify variables independently associated with mortality. All statistical procedures were performed using SAS 9.4 (SAS Institute Inc. Cary, NC, USA) and GraphPad Prism 5.0 (GraphPad Software Inc., La Jolla, USA). A two-tailed *P* value < 0.05 was considered statistically significant.

## Results

Table 1 shows the demographics and perioperative characteristics of the HTx recipients stratified into 3 groups by the estimated glomerular filtration rate (eGFR). Patients in the GFR < 30 ml/min/1.73 m^2^ group were older than those in the eGFR≥60 ml/min/1.73 m^2^ group (*P* < 0.001), and frequency of chronic kidney disease and the creatinine levels were higher in the GFR < 30 ml/min/1.73 m^2^ group than in the other groups (*P* = 0.0005 and *P <* 0.0001, respectively). However, there was no difference in body mass index (BMI) or left ventricular ejection fraction (LVEF) among the groups (*P* = 0.575 and 0.257, respectively). In addition, the three groups had a similar frequency of dilated cardiomyopathy (DCM), coronary artery disease (CAD), valve disease, and pre-percutaneous coronary intervention (PCI) (*P* = 0.279, 0.604, 0.756 and 0.441, respectively). Intraoperatively, patients in the GFR < 30 ml/min/1.73 m^2^ group had a longer duration of CPB and more infusion than the patients in the other groups, but there was no statistical significance (*P >* 0.05). Although there was decreased urine volume during the operation in the GFR < 30 ml/min/1.73 m^2^ group (*P* = 0.001), there was no difference in time from operation to discharge, mortality in hospital, death within 1 year, or the incidence of AKI between the groups (*P* > 0.05).

**Table 1:** Demographics and Perioperative Characteristics of the HTx Recipients Stratified by eGFR

	Overall (***n*** = 95)	eGFR (ml/min/1.73 m^**2**^)			***P*** value
		< 30 (***n*** = 25)	30–59 (***n*** = 61)	≥60 (n = 9)	
**Demographic data**					
Age, years	54.31 ± 11.92	58.88 ± 9.75	55.15 ± 9.20	35.89 ± 17.20	0.0007*
Sex, men, n (%)	81(85.26)	19(76.00)	54(88.52)	8(88.89)	0.318
BMI (kg/m^2^)	24.54 ± 3.83	23.89 ± 3.15	24.70 ± 4.08	25.25 ± 3.92	0.575
History of alcohol	29(30.53)	10(40.00)	17(27.87)	2(22.22)	0.464
History of smoking	63(66.32)	16(64.00)	41(67.21)	6(66.67)	0.960
Hypertension	39(41.05)	15(60.00)	21(34.43)	3(33.33)	0.083
Diabetes mellitus	34(35.79)	12(48.00)	19(31.15)	3(33.33)	0.334
Chronic kidney disease	20(21.05)	12(48.00)	6(9.84)	2(22.22)	0.0005*
**Pretransplant characteristics**					
DCM	38(40.00)	7(28.00)	26(42.62)	5(55.56)	0.279
CAD	27(28.42)	9(36.00)	16(26.23)	2(22.22)	0.604
Valve disease	15(15.79)	3(12.00)	10(16.39)	2(22.22)	0.756
Pre-PCI	11(11.58)	4(16)	7(11.48)	0(0)	0.441
ICD implantation	3(3.16)	2(8.00)	1(1.64)	0(0)	0.267
LVAD implantation	1(1.05)	0(0)	0(0)	1(11.11)	0.008*
Cardiac tumor	1(1.05)	0(0)	1(1.64)	0(0)	0.755
EF pre-HTx (%)	28(23,31)	30(24,33)	26(23,30)	25(20,30)	0.257
Creatinine (mg/dL)	1.14(0.95,1.27)	1.60(1.33,1.91)	1.10(0.95,1.22)	0.76(0.70,0.87)	<.0001*
**Intraoperative characteristics**					
CPB duration (min)	225(183,262)	225(180,270)	220(193,255)	210(180,225)	0.822
Blood transfusion (ml)	1440(1100,2100)	1400(1100,2000)	1440(1100,2200)	1640(1000,2050)	0.849
Infusion (ml)	1810(1320,2440)	2000(1505,2580)	1800(1320,2350)	1700(1190,1925)	0.483
Blood loss (ml)	1000(1000,2200)	1500(1000,2500)	1000(1000,2000)	1500(1000,2000)	0.804
Urine volume (ml)	1800(1200,2500)	1300(770,1650)	2000(1300,2500)	2150(2000,2900)	0.001*
IABP/ECMO	6(6.32)	4(16.00)	2(3.28)	0(0)	0.065
**Postoperative characteristics**					
AKI stage					
NO-AKI	62(65.26)	16(64.00)	40(65.57)	6(66.67)	0.428
Stage 1	9(9.47)	0(0)	8(13.11)	1(11.11)	
Stage 2	8(8.42)	2(8.00)	4(6.56)	2(22.22)	
Stage 3	16(16.84)	7(28.00)	9(14.75)	0(0)	
Urine volume					
1st Day after operation	2200(1980,2805)	2100(1965,2485)	2200(1980,2735)	2525(2115,3320)	0.194
2nd Day after operation	2125(1765,2505)	1935(1400,2498)	2135(1845,2500)	2425(2005,2970)	0.081
3rd Day after operation	2110(1860,2050)	2050(1400,2498)	2145(1930,2450)	2305(2050,2440)	0.300
Mechanical ventilation (min)	1080(840,2220)	1920(960,3720)	1040(783,2100)	960(720,1410)	0.134
RRT	16(16.84)	7(28.00)	9(14.75)	0(0)	0.123
Time from operation to discharge (days)	26(22,33)	29(23,37)	26(22,32)	24(20,26)	0.370
Death	15(15.79)	4(16.00)	10(16.39)	1(11.11)	0.921
Mortality in hospital	11(11.58)	4(16.00)	6(9.84)	1(11.11)	0.719
Death within 1 year	3(3.16)	0(0)	3(4.92)	0(0)	0.422
Follow-up days	608(303,1180)	555(341,951)	692(276,1180)	1451(497,2132)	0.199

* *P <* 0.05; eGFR was calculated using the Chronic Kidney Disease Epidemiology collaboration equation. *HTx* Heart transplantation, *eGFR* Estimated glomerular filtration rate, *BMI* Body mass index, *DCM* Dilated cardiomyopathy, *CAD* Coronary arterial disease, *Pre-PCI* Previous percutaneous coronary intervention, *ICD* Implantable cardioverter defibrillator, *LVAD* Left ventricular assist device, *CPB* Cardiopulmonary bypass, *RRT* Renal replacement therapy. Numbers in brackets are interquartile ranges (IQRs). ANOVA was applied to the BMI variable because of its normal distribution. The Kruskal-Wallis test was used to compare other variables

Of the 95 HTx recipients who were enrolled, 33 patients fulfilled the criteria for AKI, and 62 patients were assigned to the non-AKI group (Table 2). Although there was no difference in most perioperative variables, the CPB time, blood loss and frequency of application of intra-aortic balloon pump with venoarterial extracorporeal membrane oxygenation (IABP/ECMO) during the operation were higher in the AKI group than in the non-AKI group (*P* = 0.0149, 0.0312 and 0.0102, respectively). Moreover, the urine volume during and after the operation was lower in the AKI group (*P* < 0.005). The frequency of RRT was higher in the AKI group than in the non-AKI group (*P <* 0.0001).

**Table 2:** Demographics and Perioperative Characteristics of HTx Recipients Stratified by AKI

	AKI (***n*** = 33)	Non-AKI (***n*** = 62)	***P*** value
**Demographic data**			
Age, years	55.12 ± 12.02	53.87 ± 11.94	0.534
< 60	19(57.58)	36(58.06)	0.964
≥ 60	14(42.42)	26(41.94)	
Sex, men, n (%)	31(93.94)	50(80.65)	0.083
BMI (kg/m^2^)	25.29 ± 4.32	24.14 ± 3.51	0.164
History of alcohol	8(24.24)	21(33.87)	0.335
History of smoking	23(69.70)	40(64.52)	0.613
Hypertension	17(51.52)	22(35.48)	0.133
Diabetes mellitus	14(42.42)	20(32.26)	0.328
Chronic kidney disease	10(30.3)	10(16.13)	0.109
**Pretransplant characteristics**			
DCM	8(24.24)	30(48.39)	0.023
CAD	13(39.39)	14(22.58)	0.085
Valve disease	5(15.15)	10(16.13)	0.902
Pre-PCI	7(21.21)	4(6.45)	0.033*
ICD implantation	0(0)	3(4.84)	0.202
LVAD implantation	1(3.03)	0(0)	0.347
Cardiac tumor	0(0)	1(1.61)	0.653
EF pre-HTx (%)	28(25,31)	27(22,30)	0.359
Creatinine (mg/dL)	1.14(1,1.27)	1.14(0.95,1.27)	0.617
GFR (ml/min/1.73m^2^)	38.58(29.74,45.60)	38.05(29.68,46.23)	0.988
< 30	9(27.27)	16(25.81)	0.0986
30–59	21(63.64)	40(64.52)	
≥ 60	3(9.09)	6(9.68)	
**Intraoperative characteristics**			
CPB duration (min)	240(210,270)	209(180,240)	0.0149*
Blood transfusion (ml)	1470(1300,2000)	1440(1000,2100)	0.630
Infusion (ml)	2050(1350,2980)	1800(1300,2220)	0.108
Blood loss (ml)	1600(1000,3000)	1000(1000,2000)	0.0312*
Urine volume (ml)	1500(850,2000)	2000(1300,2700)	0.0006*
IABP/ECMO	5(15.15)	1(1.61)	0.0102*
**Postoperative characteristics**			
Urine volume			
1st Day after operation	2100(1720,2965)	2200(2030,2795)	0.223
2nd Day after operation	1850(1260,2335)	2197.5(1875,2510)	0.017*
3rd Day after operation	2015(1260,2155)	2180(2005,2615)	0.0003*
RRT	16(48.48)	0(0)	<.0001*
Mechanical ventilation (min)	1380(840–3780)	1020(780,2080)	0.109
Time from operation to discharge (days)	29(24,51)	25(22,30)	0.022*
Death	8(24.24)	7(11.29)	0.101
Mortality in hospital	7(21.21)	4(6.45)	0.038*
Death within 1 year	0(0)	3(4.84)	0.549
Follow-up days	510(258,1423)	658.5(382,1162)	0.514

* *P <* 0.05; ANOVA was applied to the BMI variable because of its normal distribution. The Kruskal-Wallis test was used to compare other variables

Table 3 shows the demographics and perioperative characteristics of 95 HTx recipients stratified into 3 groups by post-HTx RRT. Intraoperatively, the AKI with RRT group had a longer CPB time, more blood loss, lower urine volume and a higher frequency of IABP/ECMO than the other groups (*P* = 0.0088, 0.0298, 0.0021 and < 0.0001, respectively). Postoperatively, there were significant differences in urine volume, mechanical ventilation rate and mortality in hospital.

**Table 3:** Demographics and Perioperative Characteristics of HTx Recipients Stratified by RRT

	Non-AKI without RRT (n = 62)	AKI without RRT (***n*** = 17)	AKI with RRT (***n*** = 16)	***P*** value
**Demographic data**				
Age, years	53.87 ± 11.94	52.24 ± 14.63	58.19 ± 7.77	0.597
< 60	36(58.06)	10(58.82)	9(56.25)	0.988
≥ 60	26(41.94)	7(41.18)	7(43.75)	
Sex, men, n (%)	5(80.65)	17(100)	14(87.50)	0.135
BMI (kg/m^2^)	24.14 ± 3.51	25.11 ± 4.52	25.47 ± 4.23	0.368
History of alcohol	21(33.87)	2(11.76)	6(37.50)	0.176
History of smoking	40(64.52)	13(76.47)	10(62.50)	0.616
Hypertension	22(35.48)	9(52.94)	8(50.00)	0.318
Diabetes mellitus	20(32.26)	6(35.29)	8(50.00)	0.422
Chronic kidney disease	10(16.13)	3(16.13)	7(43.75)	0.052
**Pretransplant characteristics**				
DCM	30(48.39)	5(29.41)	3(18.75)	0.062
CAD	14(22.58)	5(29.41)	8(50.00)	0.097
Valve disease	10(16.13)	2(11.76)	3(18.75)	0.854
Pre-PCI	4(6.45)	5(29.41)	2(12.50)	0.033*
ICD implantation	3(4.84)	0(0)	0(0)	0.442
LVAD implantation	0(0)	1(5.88)	0(0)	0.098
Cardiac tumor	1(1.61)	0(0)	0(0)	0.764
EF pre-HTx (%)	27(22,30)	28(25,30)	28.5(24.5,33.5)	0.571
Creatinine (mg/dL)	1.14(0.95,1.27)	1.14(1.00,1.19)	1.25(1.04,1.51)	0.244
GFR (ml/min/1.73 m^2^)	38.05(29.68,46.23)	39.18(35.39,47.80)	37.36(24.10,40.43)	0.183
< 30	16(25.81)	2(11.76)	7(43.75)	0.189
30–59	40(64.52)	12(70.59)	9(56.25)	
≥ 60	6(9.68)	3(17.65)	0(0)	
**Intraoperative characteristics**				
CPB duration (min)	209(180,240)	210(210,240)	269(232.5297.5)	0.0088*
Blood transfusion (ml)	1440(1000,2100)	1440(1000,1812)	1500(1300,2625)	0.499
Infusion (ml)	1800(1300,2220)	1980(1400,2950)	2055(1285,3025)	0.275
Blood loss (ml)	1000(1000,2000)	1000(1000,2500)	2200(1300,3350)	0.0298*
Urine volume (ml)	2000(1300,2700)	1500(1000,2000)	1100(710,2000)	0.0021*
IABP/ECMO	1(1.61)	0(0)	5(31.25)	<.0001*
**Postoperative characteristics**				
Urine volume				
1st Day after operation	2200(2030,2795)	2470(2100,3010)	1597.5(521,2214)	0.0016*
2nd Day after operation	2197.5(1875)	2130(1800,2465)	1253.5(145,2087.5)	0.0020*
3rd Day after operation	2180(2005,2615)	2090(2000,2265)	1365(75,2112.5)	0.0002*
Mechanical ventilation (min)	1020(780,2080)	960(783,1380)	3090(1550,6141)	0.0033*
Time from operation to discharge (days)	25(22,30)	28(24,34)	38(24.5,64)	0.029*
Death	7(11.29)	0(0)	8(50.00)	0.0001*
Mortality in hospital	4(6.45)	0(0)	7(43.75)	<.0001*
Death within 1 year	3(4.84)	0(0)	0(0)	0.438
Follow-up days	658.5(382,1162)	954(489,2336)	261(24.5736.5)	0.0028*

* *P <* 0.05; ANOVA was applied to the BMI variable because of its normal distribution. The Kruskal-Wallis test was used to compare other variables

The overall hospital mortality was 11.58%(11/95). The orrcurence of AKI was associated with mortality in hospital (*P* = 0.0383). Patients requiring RRT had a hospital mortality of 43.75% (7/16) compared with 6.45% (4/62) in those patients without AKI or RRT (Fig. 3 and Table 3). The causes of death were as follows: sepsis (*n* = 3), respiratory failure (*n* = 2), cerebral hemorrhage (*n* = 1), pulmonary embolism (n = 1), gastrointestinal bleeding (n = 1), disseminated intravascular coagulation (n = 1), shock (n = 1), and 1 patients without diagnosis. Multivariate logistic regression analysis suggested that AKI requiring RRT was a risk factor independently associated with hospital mortality (Table 4).

**Table 4:** Univariate and multivariate analysis of characteristics associated with in-hospital mortality

	OR	95% CI		***P*** value
		Lower-limit	Upper-limit	
**Univariate**				
Age, years ≥60	4.333	1.071	17.534	0.0398*
CPB duration	1.005	0.999	1.010	0.0940
Blood loss	1.000	0.999	1.000	0.683
Urine volume	1.000	0.999	1.000	0.247
IABP/ECMO	10.125	1.746	58.700	0.0098*
AKI	3.904	1.051	14.507	0.042*
AKI requiring RRT	11.278	2.740	46.424	0.0008*
**Multivariate**				
AKI requiring RRT (ref = no-AKI and no-RRT)	11.348	2.418	53.267	0.002*
IABP/ECMO	2.302	0.299	17.743	0.424

* P < 0.05; *OR* Odds ratio, *CI* Confidence interval, *CPB* Cardiopulmonary bypass, *IABP* Intra-aortic balloon pump, *ECMO* Extracorporeal membrane oxygenation, *AKI* Acute kidney injury, *RRT* Renal replacement therapy

**Fig. 1:**
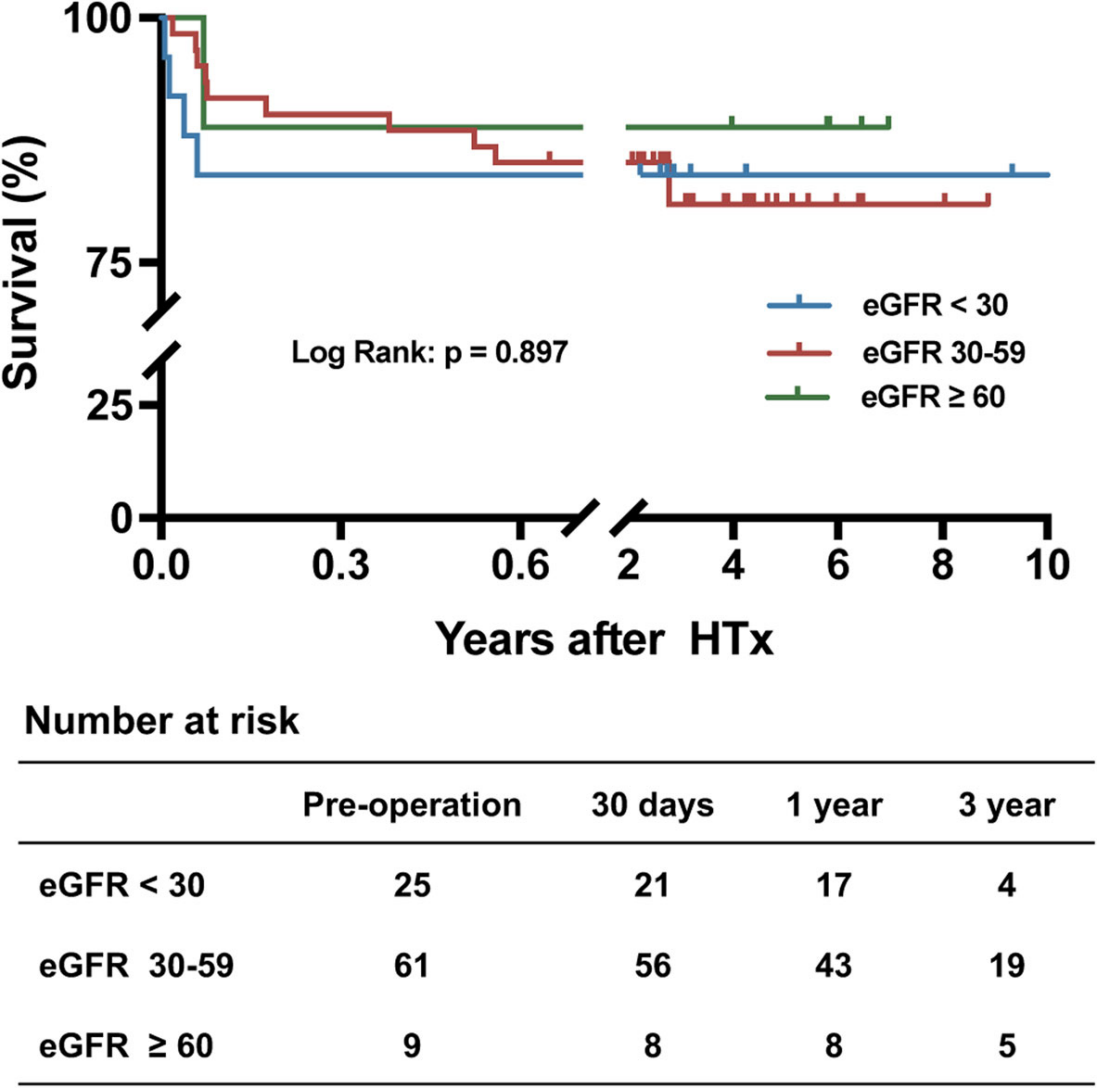
Kaplan-Meier curves for overall survival. Analysis stratified by eGFR. eGFR, estimated glomerular filtration rate in ml/min/1.73 m^2^

**Fig. 2:**
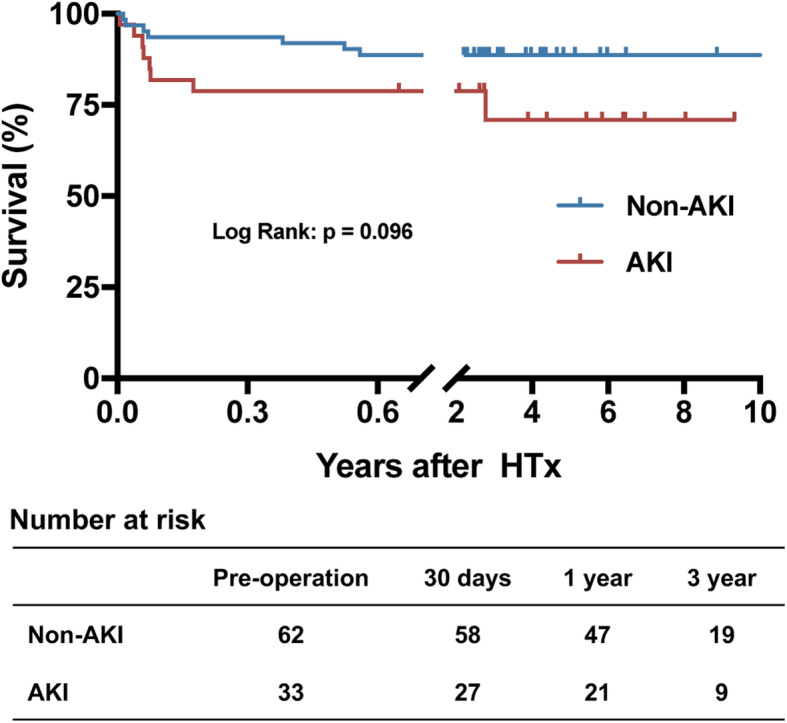
Kaplan-Meier curves for overall survival. Analysis stratified by AKI. AKI, acute kidney injury

**Fig. 3:**
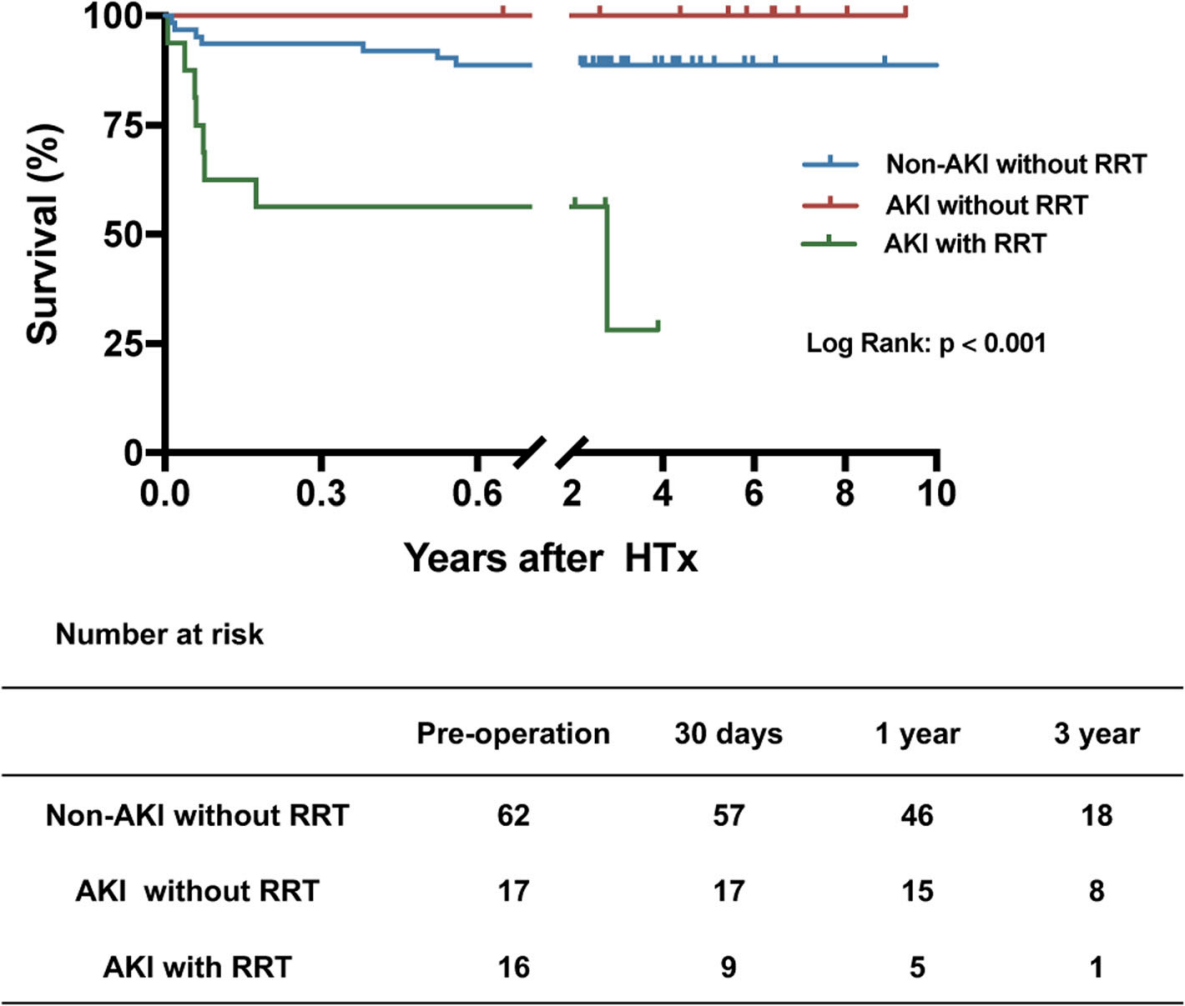
Kaplan-Meier curves for overall survival. Analysis stratified by RRT. RRT, renal replacement therapy

The median duration of follow-up after hospital discharge was 608 days (interquartile range, 303–1180 days) with a maximum of 3405 days. The clinical outcomes are summarized by eGFR stratification in Fig. 1. There were no differences in long-term survival among the 3 groups stratified by eGFR (*P* = 0.897). When stratified by AKI, there was no difference in the overall 3-year survival rates (*P* = 0.096) (Fig. 2). However, the survival rate was 72.16 ± 16.38% in the AKI with RRT group, while the survival rate was 89.43 ± 2.33% in the non-AKI without RRT group (*P* < 0.001) (Fig. 3). A total of 4 deaths were observed during the follow-up period. The causes of death within 1 year were as follows: sepsis (*n* = 2) and tumor metastasis (*n* = 1). One patient requiring RRT passed away due to cerebral infarction at 3 years after HTx.

The multivariable model for AKI is summarized in Table 5. When the CPB time is more than 265 min, it was positively related to the occurrence of AKI (OR: 11.393, 95% CI: 2.183 to 59.465, *P* = 0.0039). A decreased intraoperative urine volume, less than 1700 ml, it may positively correlated with AKI (OR: 0.181, 95% CI: 0.042 to 0.774, *P* = 0.0211).

**Table 5:** Multivariate model for AKI

	N(%)	OR	95% CI		***P*** value
			Lower-limit	Upper-limit	
CPB duration					
< 195(ref)	26 (27.37)	1	NA	NA	NA
195–225	31 (32.63)	1.853	0.477	7.206	0.373
226–265	15 (15.79)	3.674	0.724	18.638	0.116
≥ 265	23 (24.21)	11.393	2.183	59.465	0.0039*
Urine during operation					
< 1200(ref)	22 (23.16)	1	NA	NA	NA
1200–1700	25 (26.32)	0.181	0.042	0.774	0.0211*
1701–2300	23 (24.21)	0.514	0.143	1.853	0.309
≥ 2300	25 (26.32)	0.031	0.005	0.212	0.0004*

* *P <* 0.05; *OR* Odds ratio, *CI* Confidence interval, *CPB* Cardiopulmonary bypass, *NA* Not applicable, *HR* Hazard ratio, *AKI* Acute kidney injury, *RRT* Renal replacement therapy

## Discussion

In our retrospective analysis, we found that AKI was a frequent complication of HTx, with an incidence of 34.7%. We also showed that a relatively short CPB time (≤ 265 min) and increased intraoperative urine volume could prevent the occurrence of AKI. Furthermore, AKI requiring RRT was an independent risk factor for in-hospital mortality after HTx. Finally, AKI requiring RRT was not associated with long-term mortality.

Severe AKI is an important independent contributor to mortality in the HTx population. Accumulating evidence indicates that AKI requiring RRT could be a strong predictor of adverse clinical outcomes. In Renata’s study, patients with AKI, especially those requiring RRT (46.9%), had higher hospital mortality (16%) than those without AKI [14]. However, after hospital discharge, AKI was not associated with poor long-term outcomes. With a median follow-up after hospital discharge of 6.7 years, overall survival at 1, 5, and 10 years was 95.4, 85.1, and 75.4% and 85.2, 69.8 and 63.5% among patients with AKI stages 2 and 3, respectively [14]. Fortrie’s findings showed that one-year mortality rates in patients without AKI and with AKI stages 1, 2, and 3 were 4.8, 7.6, 11.8, and 14.7%, respectively [7]. In an extensive follow-up of 471 HTx patients over a period up to 26 years, no association was found between the development of AKI and long-term mortality or chronic RRT dependence [15]. In this study, we found that mortality in hospital in patients with AKI was 21.21%, and the incidence rate of AKI requiring RRT was 48.48%. Moreover, overall survival in patients without AKI at 1, and 3 years was higher than that in AKI patients.

In contrast to the high overall incidence of AKI, the need for RRT in our study was 16.84%. This is similar to previous studies reporting a need for RRT in 6 to 29% of patients [2, 4, 6]. A recent analysis indicated that AKI requiring RRT had a 1-year mortality rate of 59.2% [16]. In Boyle’s study, AKI requiring RRT was associated with a mortality rate of 50% compared to 1.4% in patients without AKI [17]. We estimated an increased risk for in hospital mortality, with an odd ratio of 11.348 in AKI patients requiring RRT. These results could be explained by the fact that patients with severe AKI are less likely to achieve full recovery of kidney function, even with RRT, than patients with mild AKI. In fact, some AKI patients requiring RRT develop at least one other serious complication (sepsis, graft failure, or acute myocardial infarction), which can lead to early mortality during the postoperative care period. In our study, seven patients in AKI with RRT group passed away in hospital. However, there was a nonsignificant tendency toward an increase in long-term mortality in AKI patients requiring RRT, which is consistent with previous reports [3]. Therefore, the impact of RRT appears to be lost at long-term follow-up. This result indicated that recovery of kidney function prior to hospital discharge was associated with decreased long-term mortality risk.

The interactions between the heart and kidney systems have become a matter of great concern [18]. The difference between arterial driving pressure and venous outflow pressure must remain sufficiently large for adequate renal blood flow and glomerular filtration. The low-resistance nature of the renal vasculature and parenchyma and the very low oxygen tension in the outer medulla also explain the unique sensitivity of the kidneys to hypotension-induced injury [2, 19]. Thus, both hemodynamic instability and antecedent hypotension should be considered in the consultative evaluation of a patient with developing AKI.

Several factors have been suggested to contribute to the development of postoperative AKI. In general, the most common cause in the early postoperative period is ischemic-reperfusion injury [20]. Intraoperatively, maintenance of a mean arterial pressure (MAP) > 60–65 mmHg, reduction in CPB time, minimization of blood transfusion and avoidance of nephrotoxic agents may prevent AKI [2, 21]. Moreover, increased central venous pressure (CVP) was associated with a reduced GFR and all-cause mortality. Right atrial pressure strongly predicts the development of AKI early after HTx and can be used as an early AKI indicator [22]. Finally, postoperatively, chloride-restricted fluid management was associated with less AKI and RRT [23]. In our opinion, a relatively short CPB time and increased intraoperative urine volume play important roles in preventing the occurrence of AKI after HTx.

When AKI occurs, the most important thing is the time of applying RRT. It offers steady fluid removal and their intensity can be easily titrated for prevention or rapid administration of treatment of volume overload. This intervention in the postoperative management can prevent a higher progression of perioperative AKI, and the occurrence of the worst outcomes [24].

Although left ventricular assist device (LVAD) is widely applied as a bridge to HTx, kidney dysfunction is common after LVAD implantation. In theory, improvements in cardiac output after implantation of LVAD would be expected to improve renal perfusion. Previous studies shown that the improvement in renal function was seen during the first month postimplantation of LVAD and no further improvements occurred thereafter [25, 26]. However, a decrease in renal function after implantation raising uncertainties about the long-term effects of continuous blood flow on renal function [27, 28]. The incidence of postimplantation AKI is 7–14% in continuous-flow devices [29]. In addition, RRT is needed in a subset of patients who develop post-LVAD AKI [30]. In our study, a patient with non-pulsatile flow LVAD implantation developed AKI stage 2 before HTx but there is no AKI in post-HTx. We consider that the influence of LVAD on kidney do not affect the treatment effect of HTx.

The performance and usefulness of different AKI scoring systems with regard to mortality vary greatly [31]. The KDIGO criteria are widely applied in the analysis of AKI in HTx patients. However, the emphasis on SCr and urine volume may exaggerate the severity of AKI. In addition, according to the RIFLE criteria, AKI encompasses the entire spectrum of the syndrome, from minor changes in renal function to the requirement of RRT. Thus, AKI does not simply represent acute renal failure but is a more general description [32]. Since the AKIN criteria are not sensitive enough to capture all episodes of AKI in cardiac surgery patients, they are not widely used for HTx patients [33]. We consider to evaluate this issue in future clinical trials.

We acknowledge that several limitations exist in this study. The inherent limitation is that it was a retrospective, single-center study that enrolled a small number of patients. Furthermore, the small sample size made it difficult to detect small effects and prevented the accuracy of multivariate analysis. In addition, patients were relatively old and likely to suffer from comorbid conditions, such as diabetes mellitus and hypertension. These comorbidities may interfere with the analysis of the long-term survival rates in AKI requiring RRT. Finally, the indication for dialysis is standardized; however, to some extent, it depends on the physician treating the individual patient, which may have acted as a confounder in our study.

## Conclusions

Our study suggests that AKI is a frequent complication of HTx, and the results demonstrated that AKI requiring RRT following HTx was associated with an increased risk for for in-hospital mortality. However, AKI patients had a relatively good long-term prognosis, with the recovery of renal function. Therefore, the results of this study highlight that risk factor identification may assist in implementing strategies to prevent or limit the progression of AKI, which, in turn, may improve survival.

## Data Availability

The datasets used and/or analyzed during the current study are available from the corresponding author on reasonable request.

## Abbreviations

AKI: Acute kidney injury
HTx: Heart transplantation;
KDIGO: Kidney Disease: Improving Global Outcomes
RRT: Renal replacement therapy
CPB: Cardiopulmonary bypass
RIFLEL: Risk/Injury/Failure/Loss/End-stage
AKIN: Acute Kidney Injury Network
SCr: Serum creatinine
ANOVA: One-way analysis of covariance
eGFR: estimated glomerular filtration rate
BMI: Body mass index
LVAD: Left ventricular assist device
LVEF: Left ventricular ejection fraction
DCM: Dilated cardiomyopathy
CAD: Coronary artery disease
PCI: percutaneous coronary intervention
IABP: Intra-aortic balloon pump
ECMO: Extracorporeal membrane oxygenation
OR: Odds ratio
CI: Confidence interval
